# Molecular basis of human poly(A) polymerase recruitment by mPSF

**DOI:** 10.1261/rna.079915.123

**Published:** 2024-07

**Authors:** Sofia Todesca, Felix Sandmeir, Achim Keidel, Elena Conti

**Affiliations:** Department of Structural Cell Biology, Max Planck Institute of Biochemistry, 82152 Martinsried, Germany

**Keywords:** 3′ end processing, 3′ end formation, polyadenylation, PAPOA, pre-mRNA, cleavage and polyadenylation

## Abstract

3′ end processing of most eukaryotic precursor-mRNAs (pre-mRNAs) is a crucial cotranscriptional process that generally involves the cleavage and polyadenylation of the precursor transcripts. Within the human 3′ end processing machinery, the four-subunit mammalian polyadenylation specificity factor (mPSF) recognizes the polyadenylation signal (PAS) in the pre-mRNA and recruits the poly(A) polymerase α (PAPOA) to it. To shed light on the molecular mechanisms of PAPOA recruitment to mPSF, we used a combination of cryogenic-electron microscopy (cryo-EM) single-particle analysis, computational structure prediction, and in vitro biochemistry to reveal an intricate interaction network. A short linear motif in the mPSF subunit FIP1 interacts with the structured core of human PAPOA, with a binding mode that is evolutionarily conserved from yeast to human. In higher eukaryotes, however, PAPOA contains a conserved C-terminal motif that can interact intramolecularly with the same residues of the PAPOA structured core used to bind FIP1. Interestingly, using biochemical assay and cryo-EM structural analysis, we found that the PAPOA C-terminal motif can also directly interact with mPSF at the subunit CPSF160. These results show that PAPOA recruitment to mPSF is mediated by two distinct intermolecular connections and further suggest the presence of mutually exclusive interactions in the regulation of 3′ end processing.

## INTRODUCTION

Eukaryotic precursor-mRNAs (pre-mRNAs) are extensively processed during transcription in the nucleus to generate mature mRNAs that are then exported into the cytoplasm (Hsin and Manley 2012; Vorländer et al. 2022). The 3′ ends of most mRNAs are formed by an essential two-step reaction, cleavage and polyadenylation, in which the pre-mRNAs are endonucleolytically cleaved at a specific site before the addition of a stretch of adenine nucleotides [poly(A) tail] (Kumar et al. 2019; Sun et al. 2020; Boreikaitė and Passmore 2023). The cleavage and polyadenylation process is crucial for both terminating RNA polymerase II transcription and for regulating various aspects of the mRNA life cycle, including mRNA stability and translation (Proudfoot 2016; Eaton and West 2020; Passmore and Coller 2022).

In human cells, 3′ end processing hinges on the orchestrated assembly of multiple protein modules at specific sequence elements present at the 3′ end of the pre-mRNA. Central to these modules is the mammalian polyadenylation specificity factor (mPSF). The mPSF is responsible for recognizing the canonical polyadenylation signal (PAS), a short consensus motif (often AAUAAA) (Proudfoot and Brownlee 1976; Shi et al. 2009; Sun et al. 2020). After recognition of the PAS, the mPSF complex coordinates additional factors necessary for the catalytic steps (Chan et al. 2014; Schönemann et al. 2014; Clerici et al. 2018; Sun et al. 2018). In particular, the endonucleolytic cleavage step requires the mammalian cleavage factor (mCF, which contains the endonuclease CPSF73), the cleavage stimulation factor (CstF), the cleavage factors I and II (CF-I and CF-II), the retinoblastoma-binding protein 6 (RBBP6), and poly(A) polymerase α (PAPOA) (Kumar et al. 2019; Sun et al. 2020; Boreikaite et al. 2022; Schmidt et al. 2022). Many of these factors exhibit a high degree of conservation across mammals and yeast, emphasizing the importance of the process (Xiang et al. 2014). The regulated interplay of these proteins alongside auxiliary factors can also modulate cleavage site selection and processing efficiencies in vivo (Tian and Manley 2017; Gruber and Zavolan 2019).

The subsequent polyadenylation of the cleaved pre-mRNA results from PAPOA activity (Wahle 1991a; Bienroth et al. 1993; Schönemann et al. 2014). PAPOA contains a structured core at the N terminus that harbors the enzymatic activity and that consists of catalytic, central, and RNA-binding (RBD) domains (Wahle 1991b; Martin and Keller 1996; Martin et al. 2000). Human PAPOA also contains an extended C terminus that is known to be posttranslationally modified (Colgan et al. 1996; Kim et al. 2003; Vethantham et al. 2008). Although the polymerase activity is rather weak and distributive when PAPOA is in isolation, it greatly increases by interacting with mPSF and the nuclear poly(A)-binding protein PABPN1 (Wahle 1991a,b; Bienroth et al. 1993; Schönemann et al. 2014).

mPSF consists of four subunits, CPSF160, WDR33, CPSF30, and FIP1, and serves as a scaffold module to recruit the catalytically active proteins CPSF73 and PAPOA to the pre-mRNA substrate (Chan et al. 2014; Schönemann et al. 2014; Zhang et al. 2020). CPSF160 contains three β-propeller domains (BPA, BPB, and BPC), which are ordered in a trefoil arrangement, and recruits WDR33 and CPSF30 for high-affinity binding to the RNA. The PAS is recognized by the N-terminal WD40 domain of WDR33 and by two zinc fingers of CPSF30 (zinc fingers 2 and 3) that become ordered upon interacting with the RNA (Clerici et al. 2018; Sun et al. 2018). FIP1 is a protein predicted to be mostly disordered and bridges CPSF30 and PAPOA, thereby tethering mPSF to PAPOA during poly(A) tail synthesis (Kaufmann et al. 2004; Hamilton and Tong 2020; Muckenfuss et al. 2022). Structural studies have previously shown that the yeast ortholog Pap1 is bound by a short Fip1 motif onto the outward-facing surface of the Pap1 RBD (Meinke et al. 2008). However, the Fip1 sequence is highly divergent, and residues at the interaction surface of both Fip1 and Pap1 are poorly conserved between yeast and human (Meinke et al. 2008; Muckenfuss et al. 2022). In addition, an earlier study reported that human PAPOA can also interact with the mPSF subunit CPSF160 (Murthy and Manley 1995).

Overall, the recruitment of human PAPOA to its mPSF-bound pre-mRNA substrates is a critical step during 3′ end formation, yet the underlying molecular and structural basis is currently not fully understood. Using a combination of structural and biochemical methods, we show the mechanisms with which both the structured and unstructured domains of PAPOA engage the mPSF complex in a bidentate mode of interactions, with the FIP1 and CPSF160 subunits, respectively. The results not only provide insights into the recruitment of human PAPOA to mPSF, but also suggest a potential regulatory mechanism.

## RESULTS

### Identification of the PAPOA-binding region of FIP1

**FIGURE 1.**
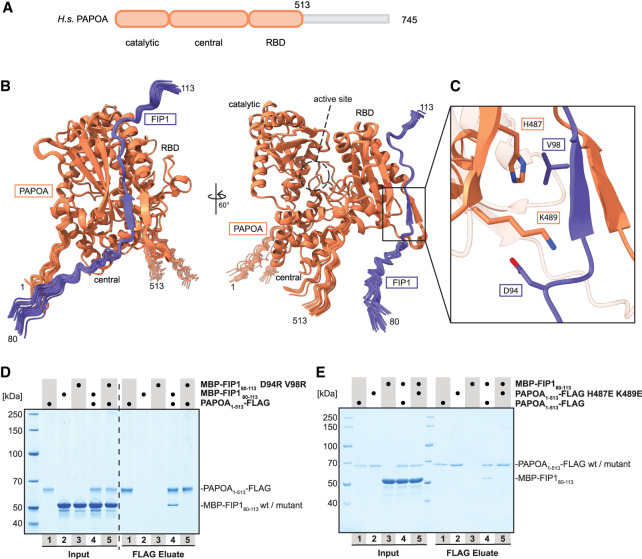
Identification of the PAPOA-binding region of FIP1. (*A*) Domain organization of human PAPOA. The truncated construct used here is indicated in orange. (*B*) Overlay of 25 computationally predicted models of the PAPOA_1–513_–FIP1_80–113_ complex, shown in two orientations. The PAPOA structured domains and active sites are indicated. (*C*) Close-up of the putative binding interface. Labeled residues were changed to arginine (FIP1) or glutamic acid (PAPOA). (*D*) Coomassie-stained SDS-PAGE analysis of pull-down experiment showing that positively charged FIP1 mutations in the predicted PAPOA–FIP1 interface disrupt the interaction. (*E*) Coomassie-stained SDS-PAGE analysis of pull-down experiment showing that negatively charged PAPOA mutations in the predicted PAPOA–FIP1 interface disrupt the interaction.

To identify a minimal human FIP1 construct sufficient for the interaction with PAPOA, we performed in vitro pull-down experiments using purified proteins. The FIP1 N terminus (residues 1–113) was copurified with the structured core of PAPOA (PAPOA core; residues 1–513; Fig. 1A; Supplemental Fig. S1A), as reported previously (Kaufmann et al. 2004). Because the precise binding region is currently unclear, we used AlphaFold2 (Jumper et al. 2021) to computationally predict structural models of the PAPOA core–FIP1 interaction site.

In these predictions, the PAPOA core adopts a U-shaped arrangement with the three folded domains surrounding the catalytic center, very similar to the reported structures of bovine and yeast poly(A) polymerases (Fig. 1B; Bard et al. 2000; Martin et al. 2000). After comparing multiple predictions, only an extended FIP1 peptide (residues 80–113; 25/25 models) reproducibly docked onto the outward-facing side of the PAPOA RBD, opposite to the active site, without blocking access to the substrate-binding cleft (Fig. 1B). FIP1 aligns into a concave surface of the PAPOA core, driven by a combination of side chain– and main chain–mediated interactions. In particular, FIP1 (amino acids 96–99) and PAPOA (amino acids 452–455) form two parallel β-strands (Fig. 1C). Interestingly, the FIP1 N-terminal residues (1–79) adopt highly heterogeneous conformations in space, suggesting that only the short peptide motif anchors FIP1 onto the surface of PAPOA (Supplemental Fig. S1B).

To test our model experimentally, we purified a minimal FIP1 construct (residues 80–113), which was sufficient for the interaction with PAPOA in pull-down assays (Fig. 1D,E). To validate the putative binding site, we introduced charged mutations either in the minimal FIP1 peptide (D94R V98R) or the structured PAPOA core (H487E K489E), which completely disrupted the interaction between the proteins (Fig. 1C–E). Moreover, these findings were recapitulated when full-length poly(A) polymerase and a longer FIP1 construct (residues 1–393) were expressed in mammalian cells. Coprecipitation experiments using HEK cell lysates confirmed that the same FIP1-charged mutations abolished the formation of the complex, verifying that the identified interface represents the main binding epitope between PAPOA and FIP1 (Supplemental Fig. S1C).

In summary, we identified a minimal FIP1 peptide (residues 80–113) sufficient for the interaction with PAPOA. In general, despite relatively poor sequence conservation between yeast and human, both species exploit an overall similar binding mode, using a FIP1 peptide motif docking onto the outer side of the RBD to recruit the poly(A) polymerase (Supplemental Fig. S1D–G; Meinke et al. 2008).

### FIP1 and PAPOA C terminus compete for binding to the PAPOA RNA-binding domain

**FIGURE 2.**
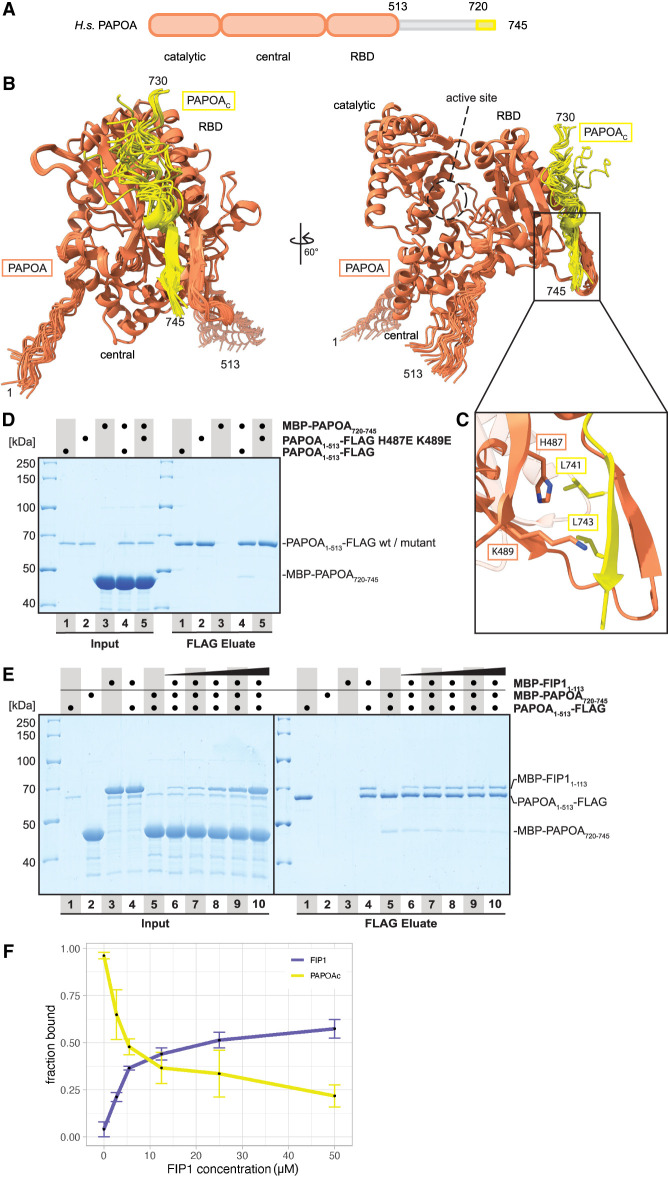
PAPOA_C_ and FIP1 compete for binding to the PAPOA RNA-binding domain. (*A*) Domain organization of human PAPOA. The truncated constructs used here are indicated in orange (PAPOA structured core) or yellow (PAPOA_C_). (*B*) Overlay of 25 computationally predicted models of the PAPOA_1–513_–PAPOA_C_ complex shown in two orientations (PAPOA_C_ residues 720–729 are not shown for clarity). The PAPOA structured domains and active sites are indicated. (*C*) Close-up of the putative binding interface. Labeled residues of the PAPOA core were changed to glutamic acid. (*D*) Coomassie-stained SDS-PAGE analysis of pull-down experiment showing that negatively charged PAPOA mutations in the predicted PAPOA–PAPOA_C_ interface disrupt the interaction. (*E*) Coomassie-stained SDS-PAGE analysis of competition pull-down experiment showing that PAPOA_C_ and FIP1 compete for the same binding surface on the PAPOA structured core. (*F*) Quantification of the competition pull-down experiment performed in triplicates.

The PAPOA C terminus (PAPOA_C_) has been shown to serve as a binding platform facilitating the regulation of poly(A) polymerase activity by splicing factors (Gunderson et al. 1997, 1998; Ko and Gunderson 2002). Interestingly, AlphaFold2 predictions of full-length PAPOA suggest that the C terminus may serve yet another purpose (Varadi et al. 2022). In fact, multiple predictions reproducibly align a peptide motif of PAPOA_C_ (residues 730–745; 25/25 models) into the concave surface on the outward-facing side of the PAPOA RBD, thereby forming an antiparallel β-strand with PAPOA amino acids 452–455 (Fig. 2A–C). Notably, these PAPOA_C_ residues (in particular L741, L743) interact with the RBD by docking exactly onto the surface of the PAPOA structured core where FIP1 binds (Figs. 1C and 2A,B; Supplemental Fig. S2A–C; Rodríguez-Molina and Turtola 2023). To test these predictions experimentally, we purified a minimal PAPOA_C_ construct (residues 720–745) for pull-down assays with the structured core of PAPOA. Using low salt conditions (50 mM NaCl) and a large excess of prey protein (40× excess), we could repeatedly copurify the PAPOA_C_ with its structured core (Fig. 2D). To validate the putative binding interface, we tested the PAPOA-charged mutations (H487E K489E) that had completely abolished the interaction with FIP1 (Figs. 1E and 2C). Similarly, introducing these amino acid substitutions severely impaired the PAPOA core–PAPOA_C_ interaction (Fig. 2D). We conclude that the PAPOA_C_ can directly bind to the same outward-facing RBD surface as FIP1.

As both FIP1 and PAPOA_C_ dock onto the same PAPOA RBD surface, we used competition assays to assess whether the binding of one peptide motif would preclude the recruitment of the other one due to steric hindrance. After prebinding PAPOA_C_ to the PAPOA core, we added increasing amounts of FIP1 (residues 1–113) to the reaction mix (Fig. 2E). As expected, FIP1 titration leads to a progressive loss of PAPOA_C_ binding, confirming the mutually exclusive interaction. Even the lowest FIP1 concentration (2.75 µM FIP1 and 100 µM PAPOA_C_) could visibly reduce copurification of PAPOA_C_ (Fig. 2E,F). Notably, prebound FIP1 could not effectively be displaced by PAPOA_C_, even when the latter was present in large excess (data not shown). Collectively, these results are in line with earlier biochemical studies implicating a possible regulatory function of PAPOA_C_ (Gunderson et al. 1997, 1998).

### PAPOA C terminus binds the mPSF subunit CPSF160

**FIGURE 3.**
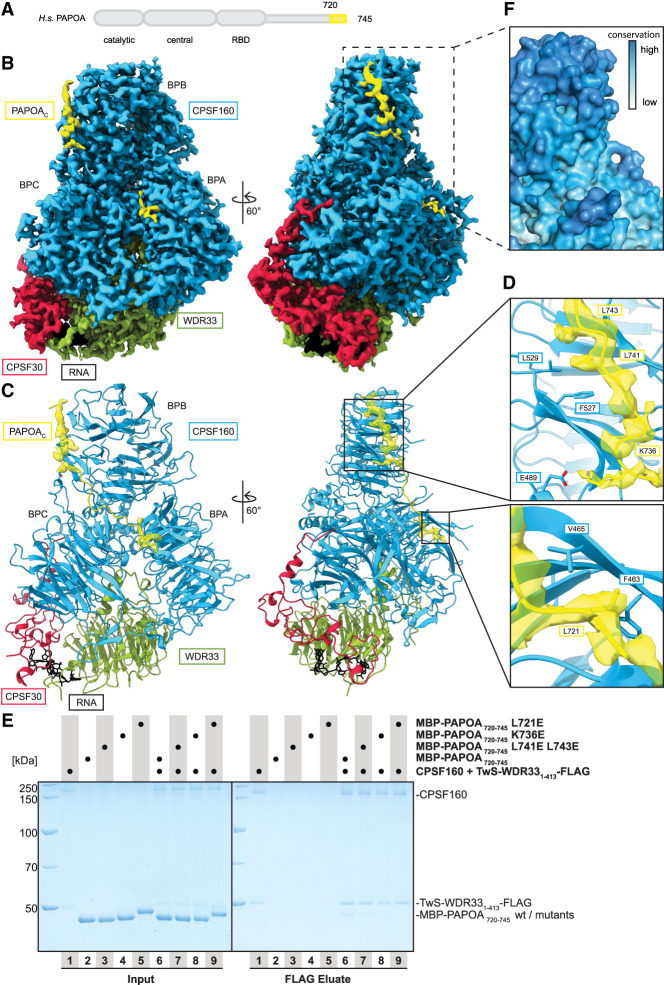
PAPOA_C_ binds the mPSF subunit CPSF160. (*A*) Domain organization of human PAPOA. The truncated construct used here is indicated in yellow. (*B*) Segmented single-particle cryo-EM reconstruction of mPSF-PAPOA_C_ at a global resolution of 2.79 Å shown in two different orientations. The densities for CPSF160, WDR33, CPSF30, PAPOA_C_, and RNA are colored in blue, green, red, yellow, and black, respectively. The CPSF160 β-propeller domains are indicated. (*C*) Structural model of mPSF overlay with the computationally predicted model of PAPOA_C_ with the segmented cryo-EM density of PAPOA_C_ displayed in transparent yellow, shown in the same orientations and colors as in *B*. (*D*) Close-up of the cryo-EM density of the binding interfaces of CPSF160–PAPOA_C_. The refined model is shown and the segmented cryo-EM density of PAPOA_C_ is displayed in transparent yellow. Labeled PAPOA_C_ residues were changed to glutamic acid. (*E*) Coomassie-stained SDS-PAGE analysis of pull-down experiment showing that the negatively charged mutations in the CPSF160–PAPOA_C_ interface impair or disrupt the interaction. (*F*) Close-up of the binding site of CPSF160 for PAPOA_C_ shown in surface representation with residues colored by sequence conservation, from low conservation in white to high conservation in blue.

Considering that PAPOA is active when bound to mPSF via FIP1 (Bienroth et al. 1993; Kaufmann et al. 2004; Eckmann et al. 2011), we asked how the PAPOA core might be released from a potential autoregulatory conformation mediated by PAPOA_C_. To address this, we ran AlphaFold2 predictions of PAPOA_C_ with the mPSF subunits CPSF160–WDR33 (Supplemental Fig. S3A), as previous data showed a direct interaction of PAPOA with CPSF160 (Murthy and Manley 1995). In these predictions, PAPOA_C_ aligns in an extended conformation onto the sides of CPSF160 β-propellers A and B (BPA and BPB), with its terminal residues docking onto BPB. To verify the computational data in the context of the mPSF complex, we mixed purified mPSF with a synthesized PAPOA_C_ peptide and subjected it to single-particle cryo-EM analysis (Supplemental Fig. S4). The final reconstruction at a global resolution of 2.79 Å was overall highly similar to previously reported reconstructions of mPSF in isolation (Fig. 3A–C; Supplemental Figs. S3B and S4; Clerici et al. 2018; Sun et al. 2018; Zhang et al. 2020). Briefly, the CPSF160 β-propeller domains form a trefoil arrangement with the N-terminal WD40 domain of WDR33 binding at the interface between BPA and BPC. CPSF30 docks onto one side of the complex, interacting with both CPSF160 BPC and WDR33, whereas the CPSF30 zinc fingers 2 and 3 are ordered because of copurification of cellular RNA (Fig. 3B,C; Supplemental Fig. S3B).

A peptide-like density docks onto the side of CPSF160 BPB, whereas an additional shorter fragment (about four residues) contacts BPA, overall matching the binding interfaces between PAPOA_C_ and CPSF160 predicted by AlphaFold2 (Fig. 3C,D; Supplemental Fig. S3A–C). However, although the predictions suggest a continuous interaction surface, the cryo-EM reconstruction shows density for two separate segments, together burying a surface area of ∼1400 Å^2^, indicating that the connecting residues are probably flexible in solution. Moreover, the PAPOA_C_ peptide density was slightly less well resolved than the other subunits, suggesting comparatively weak binding affinity between PAPOA_C_ and mPSF (Fig. 3B; Supplemental Fig. S4E). PAPOA_C_ binds to CPSF160 using a combination of ionic and hydrophobic interactions; PAPOA_C_ K736 forms a salt bridge with CPSF160 E489, whereas other PAPOA_C_ residues (L721, L741, and L743) point toward CPSF160 pockets lined with hydrophobic residues (Fig. 3D; Supplemental Fig. S3C,D). To verify the model, we introduced structure-based charged mutations at different contact sites and performed pull-down experiments using purified proteins. Indeed, these mutations impaired or disrupted the interactions between PAPOA_C_ and CPSF160–WDR33 (Fig. 3D,E; Supplemental Fig. S3C).

Taken together, the PAPOA_C_ uses the same residues (L741 and L743) to either interact in *cis* with the PAPOA structured core or in *trans* with the mPSF subunit CPSF160. Interestingly, this region is highly conserved between vertebrates, whereas it is entirely absent in yeast or variable in other invertebrates. Accordingly, the corresponding region in CPSF160 is similarly conserved across the same eukaryotic species, suggesting that functional constraints limited the evolution of the protein parts involved in the PAPOA_C_–CPSF160 interaction (Fig. 3F; Supplemental Fig. S3E,F).

## DISCUSSION

**FIGURE 4.**
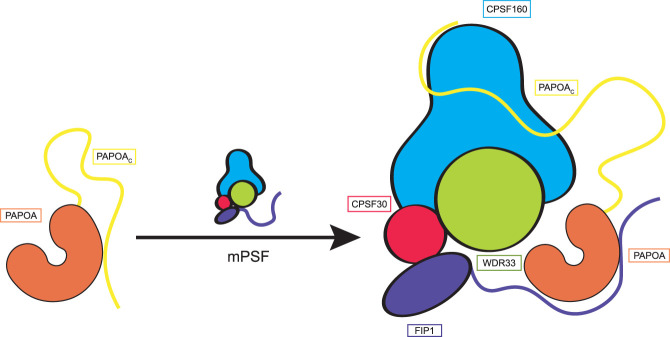
Model of PAPOA regulation. Structural and biochemical data suggest different PAPOA conformations. The PAPOA_C_ can fold back onto the PAPOA structured core in a potential autoregulatory conformation. This conformation is released in the presence of mPSF, with FIP1 docking onto the structured core and PAPOA_C_ interacting with CPSF160. The PAPOA_C_–CPSF160 interaction appears to provide a second anchoring point for tethering the poly(A) polymerase more stably to mPSF.

In this manuscript, we characterized an intricate interaction network used to recruit the human poly(A) polymerase PAPOA to mPSF. We found that PAPOA not only uses its structured core to interact with the mPSF subunit FIP1 but also engages the C-terminal unstructured region to bind to the mPSF subunit CPSF160. The PAPOA_C_–CPSF160 interaction thus appears to provide a second anchoring point for tethering the poly(A) polymerase more stably to mPSF. Intriguingly, PAPOA_C_ uses the same residues to also dock onto the PAPOA structured core, in a manner that is mutually exclusive with the docking of FIP1. Because the poly(A) polymerase is presumably FIP1-bound in the context of 3′ end polyadenylation (Bienroth et al. 1993; Kaufmann et al. 2004; Eckmann et al. 2011), a PAPOA_C_-bound state might represent an autoregulated conformation. Collectively, the data suggest a model whereby the autoregulated conformation of PAPOA is released in the presence of mPSF, with FIP1 docking onto the structured core and PAPOA_C_ interacting with CPSF160 (Fig. 4). The sequence of events is currently unclear, as additional mutually exclusive interactions and binding partners have to be taken into consideration. For example, FIP1 has been shown to bind the cleavage stimulation factor CstF77 in a mutually exclusive manner with the interaction with PAPOA, thereby inhibiting the polyadenylation activity (Muckenfuss et al. 2022). In this context, the PAPOA_C_ might be used to tether the poly(A) polymerase to mPSF during the cleavage step, thereby increasing its local concentration and facilitating a more efficient subsequent pre-mRNA polyadenylation.

These results and models have interesting implications. First, the PAPOA_C_ contains annotated sites of posttranslational modifications, such as acetylation, sumoylation, or phosphorylation. These modifications might affect the binding affinities of PAPOA_C_ toward the PAPOA structured core or CPSF160, thereby rationalizing their regulatory effect on PAPOA activity (Colgan et al. 1996; Kim et al. 2003; Vethantham et al. 2008). Second, the PAPOA_C_ sequence is necessary for the inhibition of poly(A) polymerase activity mediated by splicing factors (Gunderson et al. 1997, 1998; Ko and Gunderson 2002). It is thus tempting to speculate that PAPOA_C_ may contribute to additional layers of regulation (e.g., in the coordination between [alternative] splicing and [alternative] polyadenylation) to fine-tune the generation of different mRNA isoforms (Tian and Manley 2017; Gruber and Zavolan 2019).

In summary, the recruitment of PAPOA to mPSF before polyadenylation appears to involve a high degree of conformational and compositional flexibility. The mutually exclusive interplay of different factors, involving both their structured and unstructured domains, may impact on the sequence of events and/or fidelity with which the complex 3′ end pre-mRNA processing machinery transitions between cleavage and polyadenylation.

## MATERIALS AND METHODS

### *Escherichia coli* protein expression and purification

Proteins were expressed in *E. coli* BL21 STAR (DE3) pRARE. Bacteria were grown to an OD of 1–2 at 37°C in TB medium shaking at 190 rpm, and then the temperature was reduced to 18°C and overnight expression was induced with IPTG.

For all purifications, lysis buffers were supplemented with EDTA-free cOmplete Protease Inhibitor Cocktail (Roche), AEBSF, DNase I, and Benzonase before cells were lysed by sonication and cleared by centrifugation.

#### PAPOA

Pellets of all PAPOA structured core variants (His8-3C-PAPOA residues 1–513-FLAG, wild type or mutants) were lysed in lysis buffer (20 mM HEPES-NaOH pH 7.5, 300 mM NaCl, 10% glycerol, 20 mM imidazole, 2 mM β-ME) and purified using a Talon column (Cytiva). After extensive washing with lysis buffer and lysis buffer supplemented with 2000 mM NaCl, bound protein was eluted using a gradient by increasing the imidazole concentration to 350 mM. Fractions containing the target protein were dialyzed overnight into dialysis buffer (20 mM HEPES-NaOH pH 7.5, 150 mM NaCl, 10% glycerol, 5 mM MgCl_2_, 2 mM DTT) and diluted to 100 mM NaCl before further purification over a HiTrap Heparin column (Cytiva). After washing with wash buffer (20 mM HEPES-NaOH pH 7.5, 100 mM NaCl, 10% glycerol, 5 mM MgCl_2_, 2 mM DTT), bound protein was eluted using a gradient by increasing the salt concentration to 1000 mM NaCl, concentrated and further purified over a Superdex 200i 10/300 gel filtration column (20 mM HEPES-NaOH pH 7.5, 150 mM NaCl, 10% glycerol, 5 mM MgCl_2_, 2 mM DTT). Fractions containing pure protein were pooled, concentrated, and flash frozen in liquid nitrogen.

The PAPOA_C_ peptides (His6-MBP-3C-PAPOA residues 720–745, wildtype or mutants) were purified in MBP buffer (20 mM HEPES-NaOH pH 7.5, 150 mM NaCl, 2 mM DTT) using loose Amylose resin (Cytiva). After washing with MBP buffer and MBP buffer supplemented with 300 mM NaCl, bound protein was eluted with 25 mM maltose in MBP buffer. Eluted protein was concentrated and further purified over a Superdex 200i 10/300 gel filtration column equilibrated in MBP buffer. Fractions containing pure protein were pooled, concentrated, and flash frozen in liquid nitrogen.

#### FIP1

The FIP1 peptides (His6-MBP-3C-FIP1 residues 1–113 or 80–113, wild type or mutants) were purified in MBP buffer (20 mM HEPES-NaOH pH 7.5, 150 mM NaCl, 2 mM DTT) as described above for the PAPOA peptides.

### Insect cell protein expression and purification

#### CPSF160–WDR33

Recombinant baculovirus for coexpression of CPSF160 and TwinStrep3C-WDR33 residues 1–413-FLAG were generated in *Sf*21 cells grown at 27°C in Sf-900 II SFM medium (Thermo Fisher) using the biGBac system (Weissmann and Peters 2018). For protein expression, *Sf*9 cells grown at 27°C were infected with 1% virus solution and harvested after 3 d.

For all purifications, lysis buffers were supplemented with EDTA-free cOmplete Protease Inhibitor Cocktail (Roche), AEBSF, DNase I, and Benzonase before cells were lysed by sonication and cleared by centrifugation.

CPSF160–WDR33 were purified in buffer A (20 mM Tris-HCl pH 7.5, 150 mM NaCl, 10% glycerol, 2 mM DTT, 0.05% Tween-20) (adapted from Clerici et al. 2017) using a StrepTactin XT column (IBA). After extensive washing with buffer A, buffer A supplemented with 300 mM NaCl, and chaperone wash buffer (20 mM Tris-HCl pH 7.5, 150 mM NaCl, 10 mM MgCl_2_, 2 mM ATP, 10% glycerol, 2 mM DTT, 0.05% Tween-20), bound proteins were eluted with 1× BXT buffer (IBA) diluted in buffer A. Elution fractions were diluted to ∼75 mM salt using dilution buffer (20 mM HEPES-NaOH pH 7.5, 10% glycerol, 2 mM DTT) and applied to a MonoQ 5/50 GL column (Cytiva) for further purification. After washing with MonoQ buffer (20 mM HEPES-NaOH pH 7.5, 75 mM NaCl, 10% glycerol, 2 mM DTT), bound proteins were eluted using a gradient increasing the salt concentration up to 1000 mM NaCl and run over a Superdex 200i 10/300 column (Cytiva) in 20 mM HEPES-NaOH pH 7.5, 150 mM NaCl, 2 mM DTT. Fractions containing pure protein were pooled, concentrated, and flash frozen in liquid nitrogen.

### Mammalian cell protein expression and purification

#### mPSF

HEK293T cells stable expression cell lines were established using the *piggybac* transposon system by initially transfecting the cells using polyethyleneimine (Yusa et al. 2011; Li et al. 2013). Pools of cells were generated that stably expressed mPSF (TwinStrep-3C-CPSF30_isoform 2, CPSF160, WDR33 residues 1–413, FIP1 residues 1–393). For protein expression, cultures were adjusted to a density of 1 × 10^6^ cells per mL in FreeStyle 293 expression medium (Gibco, Thermo Fisher). The cells were induced with doxycycline and harvested 48 h after induction.

For all purifications, lysis buffers were supplemented with EDTA-free cOmplete Protease Inhibitor Cocktail (Roche), AEBSF, DNase I, and Benzonase before cells were lysed with a glass dounce homogenizer and cleared by centrifugation.

mPSF was purified in PBS buffer (1× DPBS supplemented with 2 mM MgCl_2_, 10 µM ZnCl_2_, 1 mM TCEP) using a StrepTrap HP column (Cytiva). After extensive washing with PBS buffer and PBS buffer supplemented with 300 mM NaCl, bound proteins were eluted with 5 mM desthiobiotin in PBS buffer. Protein preparations used for cryo-EM were concentrated and further purified over a Superose 6i 10/300 column (Cytiva) equilibrated in EM buffer [50 mM HEPES-KOH pH 7.9, 150 mM KOAc, 1 mM Mg(OAc)_2_, 1 mM TCEP]. Fractions containing pure protein were pooled, concentrated, and flash frozen in liquid nitrogen. All other protein preparations were diluted to ∼75 mM NaCl and applied to a MonoQ 5/50 GL column (Cytiva) for further purification. After washing with MonoQ buffer (20 mM HEPES-NaOH pH 7.5, 75 mM NaCl, 5 mM MgCl_2_, 10% glycerol, 10 µM ZnCl_2_, 2 mM DTT), bound proteins were eluted using a gradient increasing the salt concentration up to 1000 mM NaCl and run over a Superose 6i 10/300 column (Cytiva) in 20 mM HEPES-NaOH pH 7.9, 150 mM NaCl, 5 mM MgCl_2_, 10% glycerol, 10 µM ZnCl_2_, 2 mM DTT. Fractions containing pure protein were pooled, concentrated, and flash frozen in liquid nitrogen.

### Pull-down assays with purified proteins

#### FLAG pull-down assay

Pull-down experiments using purified proteins were performed using magnetic Protein G Dynabeads (Invitrogen, Thermo Fisher) coupled with α-FLAG M2 antibody (Sigma F1365). α-FLAG antibody was added to beads equilibrated in wash buffer (20 mM HEPES-NaOH pH 7.5, 5 mM MgCl_2_, 0.01% NP-40 substitute supplemented with either 150 mM NaCl for FIP1 pull-downs or 50 mM NaCl for PAPOA_C_ pull-downs). After incubation for 30 min rotating head-over at 4°C, beads were washed three times and used immediately.

One hundred and twenty-five picomoles of FLAG-tagged PAPOA core was mixed with 10× (FIP1) or 40× (PAPOA_C_) excess of the putative binding partner (final concentrations at 2.5 µM for PAPOA, 25 µM for FIP1, and 100 µM for PAPOA_C_) and the reaction was incubated 45 min at 4°C in wash buffer. Equilibrated beads were added and incubated 60 min rotating by inversion at 4°C. The beads were washed three times with 20× resin volume of wash buffer before bound proteins were eluted with 0.2 mg/mL 3× FLAG peptide diluted in wash buffer.

Pull-down experiments with FLAG-tagged WDR33 were performed as described above, although a wash buffer without MgCl_2_ (20 mM HEPES-NaOH pH 7.5, 150 mM NaCl, 0.01% NP-40 substitute) and less protein (75 pmol FLAG-tagged WDR33, final concentrations at 1.5 and 15 µM) was used.

#### Competition assays

Competition pull-down experiments using purified proteins were performed similarly as described above using magnetic Protein G Dynabeads (Invitrogen, Thermo Fisher) coupled with α-FLAG M2 antibody (Sigma F1365) in wash buffer (20 mM HEPES-NaOH pH 7.5, 50 mM NaCl, 5 mM MgCl_2_, 0.01% NP-40 substitute). One hundred and twenty-five picomoles of FLAG-tagged PAPOA core was mixed with 40× excess of PAPOA_C_ (final concentrations at 2.5 µM for PAPOA and 100 µM for PAPOA_C_) and the reaction was incubated 30 min at 4°C in wash buffer before increasing amounts of FIP1 were added (final concentrations at 2.75, 5.5, 12.5, 25, and 50 µM). After incubation for 45 min at 4°C, equilibrated α-FLAG coupled Protein G beads were added. Washing and elution were performed as described above. Eluates were separated on SDS-PAGE and proteins were quantified using a Gel Doc EZ imaging system and the Image Lab software (Bio-Rad). Independent triplicates were normalized to the intensities of the PAPOA core used as loading control. The fraction bound was calculated by dividing the normalized values by the intensity of the respective peptide band in the control lane (absence of the competitor). Means and standard deviations were then plotted against the FIP1 concentration present in the reaction mix.

### Pull-down assays with HEK cell lysates

For pull-down experiments using HEK cell lysate, 5 × 10^6^ cells per condition were transiently transfected using polyethyleneimine (PAPOA:FIP1 plasmid DNA ratio of 1:5), induced immediately with doxycycline, and harvested after 48 h. Flash frozen cells were thawed and lysed in wash buffer containing 20 mM HEPES-NaOH pH 7.5, 150 mM NaCl, 5 mM MgCl_2_, 0.1% NP-40 substitute, and EDTA-free cOmplete Protease Inhibitor Cocktail (Roche) was added. Automated pull-down experiments were performed using magnetic MagStrep type3 XT beads (IBA) and a KingFisher pull-down system (Thermo Fisher) operated at room temperature. After binding for 30 min, beads were washed four times before bound proteins were precipitated in the SDS-containing sample buffer.

### Cryo-EM sample preparation, data collection, and data processing

Before grid preparation, a synthesized PAPOA_C_ peptide (720—DLSDIPALPANPIPVIKNSIKLRLNR—745) was dissolved in EM buffer [50 mM HEPES-KOH pH 7.9, 150 mM KOAc, 1 mM Mg(OAc)_2_, 1 mM TCEP] supplemented with 20% DMSO. As the peptide appeared to be only partially soluble, the solution was spun down and only the supernatant was used (concentration not measurable, assumed to be 2500 µM). One micromolar of mPSF was mixed with 500× excess of peptide in EM buffer and incubated for 15 min at 4°C. Four microliters of the sample was applied onto glow-discharged Quantifoil R1.2/1.3, Cu 200 mesh grids. Grids were blotted for 3.5 sec with blot force 4 and plunge-frozen in a liquid ethane/propane mix using a Vitrobot Mark IV (Thermo Fisher) operated at 4°C and 100% humidity.

Cryo-EM data were collected on a FEI Titan Krios G2 microscope (Thermo Fisher), equipped with a “Bioquantum” postcolumn GIF (energy width 10 eV) and a Gatan K3 direct electron detector operated in counting mode. The nominal magnification during data collection was 105,000×, corresponding to a pixel size of 0.8512 Å at the specimen level. Using a beam-tilt-based multishot acquisition scheme in SerialEM (Schorb et al. 2019), the sample was imaged with a total exposure of 60.6 e^−^/Å^2^ evenly spread over 3 sec and 38 frames. The target defocus ranged between −0.6 and −2.2 µm.

Movies were preprocessed on-the-fly with MotionCor2 using Focus (Biyani et al. 2017; Zheng et al. 2017) while automatically discarding images of poor quality. Micrographs were imported into CryoSPARC v4 (Punjani et al. 2017) for CTF correction, particle picking, and further processing in 2D and 3D (Supplemental Fig. S4). Classification resulted in a final particle stack of 186,241 particles. The final 3D reconstruction was obtained from nonuniform refinement reaching a global resolution of 2.79 Å according to the gold standard FSC cut off of 0.143 (Rosenthal and Henderson 2003). During postprocessing, we accounted for per-group CTF parameters and higher-order abbreviations, as well as per particle defocus. Initial structure visualization and analysis were carried out using UCSF ChimeraX v1.6 (Pettersen et al. 2021).

### Model building and refinement

For model building, an available structural model of the mPSF complex bound to RNA (PDB: 6URO) (Zhang et al. 2020) was fitted into the 3D reconstruction using USCF ChimeraX v1.6 (Pettersen et al. 2021). The model was manually adjusted in Coot (Emsley et al. 2010). In brief, the individual chains were rigid body fitted into the map and then further improved using real-space refinement (Afonine et al. 2018). The initial model for the PAPOA_C_ peptide bound to mPSF was generated by AlphaFold2 predictions (Jumper et al. 2021). The PAPOA_C_ peptide was rigid body fitted into the 3D reconstruction followed by real-space refinement of the N terminus (residues 720–723) and the C terminus (residues 735–744). The linker between the two stretches was removed. Progress in modeling was monitored via the map-to-model correlation coefficients and map versus model FSCs (Supplemental Table S1).

## DATA DEPOSITION

The cryo-EM density map has been deposited in the Electron Microscopy Data Bank (EMDB) under the accession code EMD-19008. The model has been deposited in the Protein Data Bank (PDB) under the accession numbers 8R8R.

## SUPPLEMENTAL MATERIAL

Supplemental material is available for this article.

## COMPETING INTEREST STATEMENT

E.C. is an editor for *RNA*; all other authors declare no competing interests.

## Figures and Tables

**Sofia Todesca:**
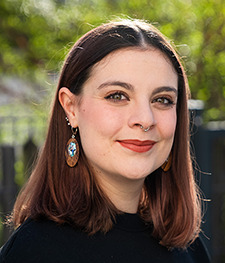


## MEET THE FIRST AUTHOR

***Meet the First Author(s)* is an editorial feature within RNA, in which the first author(s) of research-based papers in each issue have the opportunity to introduce themselves and their work to readers of *RNA* and the RNA research community. Sofia Todesca is the co-first author of this paper, “Molecular basis of human poly(A) polymerase recruitment by mPSF,” along with Felix Sandmeir. Sofia completed her bachelor and master studies in molecular biology and genetics at the University of Pavia, Italy in the laboratory of Professor Andrea Mattevi, PhD. She is now pursuing her PhD through the International Max Planck Research School for Molecules of Life program in the Department of Structural Cell Biology under the supervision of Professor Elena Conti, PhD at the Max Planck Institute of Biochemistry, Munich, Germany.**

**What are the major results described in your paper and how do they impact this branch of the field?**

Our study uncovers an intricate interaction network that governs the recruitment of human poly(A) polymerase α (PAPOA) by the mammalian polyadenylation specificity factor (mPSF). We characterized how the catalytic core of human PAPOA interacts with an unstructured region of the FIP1 subunit of mPSF, with FIP1 using a binding mode that is evolutionarily conserved from yeast to human. We discovered that the mammalian PAPOA C-terminal region folds back onto the catalytic core of PAPOA, engaging the same surface as FIP1 in a mutually exclusive manner. Intriguingly, the extended C-terminal region of PAPOA can also interact with another subunit of mPSF, CPSF160. These findings indicate that PAPOA is recruited through a dual recognition mechanism and also suggest a possible regulatory mechanism based on the presence of mutually exclusive interactions to tune polymerase activity. The emerging picture provides important insights into a critical step in human 3′ end pre-mRNA processing that leads to the synthesis of the poly(A) tail—a major determining factor to the fate of the mRNA at multiple levels, from transcript stability to translation efficiency.

**What led you to study RNA or this aspect of RNA science?**

I had never worked in the RNA field prior to beginning my PhD, but it was the synergistic pull toward the people in Elena's group and the subject matter that convinced me to join. When interviewing for my research position, I could truly recognize myself in the striving passion embodied by Elena and her group members to understand the complexity underpinning the behavior of these molecules of life, through rigorous biochemistry and structural biology methodology. My personal fascination for RNA stems from the biological complexity residing in such a dynamic molecule. It can undergo innumerable changes across its lifetime, including its spatial localization and morphological organization, secondary structures, and ribonucleoprotein associations, and these changes echo on so many different levels of the life cycle. Yet, despite this extreme variability, the basis of this molecule is shared from the simplest single-celled organism to the most complex multicellular organism.

**If you were able to give one piece of advice to your younger self, what would that be?**

I would tell my younger self to value my gut feelings more. To always question my scientific beliefs but never doubt what deep-down motivation drives me. I would remind myself to trust those small sparkles of excitement after many endeavors and to keep sharing them with people who know how to value them.

**Are there specific individuals or groups who have influenced your philosophy or approach to science?**

I am genuinely appreciative of any opportunity to name the many extraordinary people whom I have had the luck to encounter along my short scientific career. Elena Conti has been a role model in how to be a woman and scientist, and I hope to emulate her strength and determination as I continue along this path. Andrea Mattevi and Pier Paolo D'Avino believed in me since before I even considered pursuing a PhD. They taught me how important unconditional support is and how that can bring you a long way. I in turn try to support others in the same manner. From Andrea Musacchio, I learned that science is not an individual endeavor, but is most fruitful when we engage other scientists and discuss our views and ideas. I am grateful to Andrea Carfi because he reminds me that there is more than one way to interpret a life in science, and he never missed a chance to advise and mentor me. Last but not least, my unreserved gratitude goes to my university college “Collegio Ghislieri” for the boundless opportunities to learn and build my scientific acumen and much more.

**How did you decide to work together as co-first authors?**

When I joined Elena's group, Felix was nearing completion of his PhD work on 3′ end pre-mRNA processing. Felix shared his indispensable knowledge and experience, inputs, and the technical know-how to tackle the project. With this launchpad, I was able to see what we could achieve and propose and carry out the experiments that contributed to this paper. Furthermore, the discussions we had over the years have been a major driving force to the advancement of this work.
